# Regulatory Strengthening as a Pillar of Health System Resilience for Sustainable Immunization

**DOI:** 10.3390/vaccines14010033

**Published:** 2025-12-26

**Authors:** Wei Chuen Tan-Koi, Yoong Khean Khoo, John CW Lim

**Affiliations:** 1 Centre of Regulatory Excellence, Duke-NUS Medical School, Singapore 169857, Singapore; khooyk@duke-nus.edu.sg (Khoo YK); john.lim@duke-nus.edu.sg (John CW Lim); 2 Centre for Outbreak Preparedness, Duke-NUS Medical School, Singapore 169857, Singapore; 3 SingHealth Duke-NUS Global Health Institute, Duke-NUS Medical School, Singapore 169857, Singapore

**Keywords:** vaccines, regulation, regulatory strengthening, health system resilience, sustainable immunization, equitable access

## Abstract

The COVID-19 pandemic coupled with recent upheavals in global trade and development assistance funding has disrupted routine immunization programmes and diverted health systems from the targets set in the Immunization Agenda 2030. Regulatory systems are often underappreciated or misunderstood but in fact play a critical role in enabling innovation and facilitating timely access to vaccines for sustained immunization, thereby building vaccine confidence and health system resilience. Regulation is the constant denominator throughout the vaccine life cycle, shaping the pathway from early research and development to approval and market entry and ultimately to equitable distribution and sustained safe use. This paper examines the role of regulation and proposes that regulation be reframed as a function of health system resilience and a structural determinant of immunization sustainability. We synthesize evidence across the vaccine regulatory life cycle, examining innovation facilitation, regional cooperation, public health strengthening and describe the roles of regulation in building health system resilience, namely driving sustainable vaccine access, enabling innovation, supporting regional collaboration and strengthening social acceptance. Without this shift in perspective, regulatory systems strengthening risks being underfunded, reactive, and fragmented; this will perpetuate inequities in vaccine access and undermine the sustainability of immunization programmes.

## 1. Introduction

Vaccines are arguably one of the most effective public health interventions in modern times. The World Health Organization (WHO) estimates that immunization programmes prevent 4 to 5 million deaths annually worldwide [1]. From the eradication of smallpox four decades ago to the elimination of polio in many countries, the impact of vaccines in public health cannot be overstated [2]. Furthermore, immunization contributes to 14 out of 17 United Nations Sustainable Development Goals (SDGs) and directly impacts SDG3 on health and well-being [3].

The COVID-19 pandemic reminded the world of the essential importance of vaccines and immunization. The speed at which COVID-19 vaccines were developed and distributed highlighted not only the power of scientific innovation but also global commitment and solidarity. Between 2020 and 2024, these vaccines were estimated to have averted 2.5 million deaths [4]. However, the pandemic also disrupted the delivery of routine health services, including critical childhood immunization programmes. In 2021 alone, it is estimated that 25 million children worldwide missed out on one or more doses of vaccines against diphtheria, tetanus, and pertussis (DPT) [5]. This marked decline reinforces the need to tackle persistent systemic challenges while responding to emergency disruptions.

Endorsed by the 73rd World Health Assembly and formally launched in 2021, the Immunization Agenda 2030 (IA2030) sets an ambitious vision to increase access to vaccines for all segments of populations by 2030 through a strategic framework that guides immunization programmes and programmatic sustainability [6]. However, these ambitious goals have faced major setbacks with recent upheavals in global trade and significant reductions in development assistance funding which have heightened vulnerabilities in vaccine research, manufacturing and access, particularly impacting countries where health systems rely heavily on international development aid [7]. This poses significant challenges to achieving sustainable immunization in these countries.

Sustainable immunization refers to the ongoing, reliable capacity of health systems to provide routine immunizations and respond to outbreaks, ensuring long-term protection against vaccine-preventable diseases. Less visible but still critical in underpinning sustainable immunization are the regulatory systems ensuring that vaccines are safe, effective, and of high quality, especially since vaccines are administered to healthy populations. In market shaping, vaccine financing and supply have often been emphasized, while regulatory systems and governance, albeit important, are less discussed. Yet, good regulation is essential to facilitate the market access of vaccines and at the same time safeguard public trust in vaccines. While the COVID-19 pandemic highlighted the importance of good regulatory systems to facilitate access to safe vaccines, regulation has in fact long been an integral component of sustainable immunization programmes.

In this paper, we examine the role of regulation and propose that regulation be reframed as a function of health system resilience and a structural determinant of immunization sustainability. We synthesize evidence across the vaccine regulatory life cycle, examining innovation facilitation, regional cooperation, public health strengthening and present the roles of regulation in building health system resilience, namely driving sustainable vaccine access, enabling innovation, supporting regional collaboration, and strengthening social acceptance.

## 2. Regulation as a Driver for Sustainable Vaccine Access

The primary function of vaccine regulation is to ensure the quality, safety, and efficacy of vaccines. Regulation is present and relevant throughout the entire product life cycle, spanning development, pre-clinical and clinical studies, manufacturing, market authorisation, post-marketing surveillance, and compliance [8]. Good regulation aligned to international standards is essential for ensuring efficacious, safe, and quality-assured vaccines to safeguard public health and enhance trust in vaccines.

Regulatory systems strengthening to ensure sound regulation can adopt different approaches. The WHO Global Benchmarking Tool (WHO GBT) is a key international initiative that defines a standardized system for evaluating and strengthening national regulatory authorities (NRAs) for medicines, vaccines, biologics, and other health products. It benchmarks regulatory agencies across nine regulatory functions and facilitates the development of institutional development plans by NRAs to address gaps and achieve minimally well-functioning regulatory system status, i.e., maturity level (ML) 3. Achieving ML3 for countries with vaccine-producing capacity is particularly important as vaccines manufactured and approved in WHO ML3 vaccine-producing countries will be eligible for WHO’s Prequalification (PQ) programme and purchase by global procurement agencies.

The WHO PQ programme is a service provided to United Nations procurement agencies with the aim of providing qualified, safe, and efficacious medical products, including vaccines, in resource-limited settings. The programme includes evaluating how products perform, verifying that manufacturing meets global quality benchmarks, and determining whether the products are appropriate for use [9]. The vaccines PQ programme established in 1987 currently has 277 vaccines in the list [10] and plays an important role in ensuring quality-assured vaccines to support sustainable immunization programmes. This provides a critical gateway for countries with less mature regulatory functions to access global vaccine markets through international programmes such as the support provided by Gavi, the Vaccine Alliance (GAVI). However, as more countries achieve middle-income status and are no longer eligible for GAVI’s support, there is a clear need for NRAs to develop and strengthen their own regulatory frameworks and mechanisms. In addition to the WHO PQ programme, other regulatory frameworks and mechanisms such as regulatory reliance and expedited pathways could be explored to ensure timely access to vaccines. During the COVID-19 pandemic, expedited regulatory mechanisms illustrated how agile regulation could facilitate rapid access to new life-saving vaccines. A scoping review showed that regulatory pathways such as emergency use authorization, conditional marketing authorization and temporary authorization significantly reduced approval times compared to standard review times [11].

## 3. Regulation as an Enabler of Innovation

Regulation supports innovation and contributes to market sustainability by facilitating the viability of vaccine manufacturers and suppliers. Clear and predictable regulatory processes incentivize vaccine developers and manufacturers to invest in R&D and production capacity. Innovative regulatory pathways also de-risk the product development process to help encourage investment and drive innovation [12].

For example, facilitated regulatory pathways (FRPs) introduced by mature regulatory agencies, such as the European Medicines Agency (EMA), the US Food and Drug Administration (US FDA) and Japan’s Pharmaceuticals and Medical Devices Agency (PMDA), provide early engagement and regulatory guidance to developers and manufacturers, contributing to shorter development and review timelines. This reframes regulation from being viewed as a hindrance to innovation to instead providing a positive facilitatory function [13].

An example of positive facilitation to innovation is the support provided by South Korea’s Ministry of Food and Drug Safety (MFDS) for the International Vaccine Institute and EuBiologics for the oral cholera vaccine (Euvichol^®^), covering phase 1 studies, licensure and eventual WHO-prequalification within a relatively short period of three years, thereby addressing a significant global health need [14]. The rapid speed to achieve WHO PQ status had been attributed to the early engagement and support from MFDS and cited as a possible collaborative network innovation model useful to advance equitable vaccine access [15]. Another important regulatory pathway to improve vaccine access in low- and middle-income countries is the European Medicine Agency (EMA) EU-M4All which assesses innovative medicine use outside Europe [16]. An impact analysis found that following positive decisions made by EU-M4all, there were 22 subsequent approvals for DTP-HepB-IPV-Hib and 3 subsequent approvals for malaria vaccines in other countries using this route [16]. Through regulatory frameworks and mechanisms that promote reliance, agility and transparency, NRAs can support innovation and facilitate timely access to vaccines. This sustains immunization efforts and helps build up vaccine confidence which translate into health system resilience outcomes of efficiency, equity, and trust. Documenting and sharing such successful case studies would provide valuable lessons for other regulators.

## 4. Regulation as a Support for Regional Collaboration

The concept of self-reliance as a means of strengthening vaccine security and ensuring the sustainability of immunization programmes has gained traction since the COVID-19 pandemic. The African Union has a set a goal for the continent to produce 60% of its vaccine needs by 2040 with the aim of reducing external dependency on external suppliers [17]. The Pan American Health Organization (PAHO)’s Revolving Fund for Access to Vaccines has supported access to immunization in the Region of Americas since 1980 [18]. The fund is a shared pool of funds for the procurement of vaccines, syringes, and cold chain equipment. In Southeast Asia, the Association for Southeast Asian Nations (ASEAN) Vaccine Security and Self-Reliance (AVSSR) initiative, officially adopted during the 35th ASEAN Summit in 2019, identified a key strategy on procurement and regional stockpiling for vaccine accessibility in both normal and emergency scenarios [19]. In addition, the ASEAN Joint Assessment Procedure for Pharmaceutical Products, initiated in 2017, includes vaccines in the list of priority product types, allowing a single application to be submitted to multiple NRAs simultaneously. This potentially increases efficiency, fosters cooperation and reduces duplication of work among member states.

Such regional collaborative models and initiatives are predicated on the need for strong regulatory capacity through regulatory mechanisms such as reliance, convergence, and harmonization. A recent study on vaccine regulation further recommended establishing a regional Asia Pacific network to foster cooperation, enhance regulatory agility, promote reliance, strengthen regulatory capacity and facilitate good governance [8].

In establishing a regional network, it is good to foster strategic partnerships during the inter-pandemic period, when there is greater opportunity for sustained coordination and development without just reacting to socio-political and public health pressures. This can then facilitate rapid scale up during public health emergencies to cope with the likely high rate of disease spread from extensive international travel and the intense demand for essential health products. In the COVID-19 pandemic, regulators, scientists, and industries collaborated globally through mechanisms such as rolling submissions and Emergency Use Authorisation (EUA) to expedite the introduction of safe novel technologies for urgent public health needs. Sustaining this momentum through strategic partnerships during the current inter-pandemic period is critical for strengthening future pandemic regulatory readiness [20]. In doing so, regional collaboration will not only enhance emergency responsiveness but also reinforce equity, efficiency, and trust—the foundations of sustainable immunization.

However, while regional collaborative models are to be encouraged, they also carry the risk of perpetuating unhealthy dependencies if not implemented with a clear strategy for long-term capacity development. Overreliance on other agencies and other forms of technical assistance could arguably result in reduced incentives to develop national technical expertise, further widening the gap between higher-maturity and lower-maturity NRAs. Moreover, reliance mechanisms applied without sufficient contextualization may fail to fully address country specific health systems demographics and local issues, leading to inappropriate regulatory decisions. Regulatory authorities may face pressures from industry lobbying and socio-political dynamics. It is therefore important that regulatory capacity building also includes institutional training in recognizing such issues, communicating clearly, and managing stakeholders appropriately, skillfully, and wisely.

## 5. Regulation as a Key Factor to Strengthen Social Acceptance

Access to vaccines alone is not enough to ensure sustainable immunization. In this age of scientific misinformation, promoting trust and vaccine acceptance is fundamental for increasing vaccine uptake. WHO declared vaccine hesitancy as one of the top ten global health threats in 2019, highlighting the seriousness of the problem [21]. A systematic review on cognitive determinants for COVID-19 vaccines in 2022 showed one of the most common factors for vaccine hesitancy was concern about vaccine safety, effectiveness, and the rapid development and approval process [22].

Transparent, evidence-driven decision making is essential to counter misinformation and build public trust in this era of widespread vaccine hesitancy. Because safety, effectiveness, and approval are determined through defined regulatory pathways, clear and proactive communication from regulators can strengthen societal confidence, foster acceptance, and sustain high levels of vaccine uptake [23]. This was demonstrated in the COVID-19 pandemic where regulatory agencies such as the EMA and Singapore’s Health Sciences Authority (HSA) dedicated significant effort to communicating risk assessments and mitigation measures for very rare cases of myocarditis observed more often in young males following the second dose of COVID-19 vaccination [24,25].

Robust regulatory frameworks also increase equitable access to quality vaccines which are particularly critical in resource-limited settings [26]. Alignment with global benchmarking mechanisms such as the WHO GBT further reinforces confidence in the robustness of the regulatory framework and may help alleviate public concerns about the quality and safety of vaccines. However, societal trust in government and healthcare systems is complex [27]. In practice, the perceived trustworthiness of regulation and vaccine safety is also contingent on broader communication systems and the circumstances surrounding vaccines safety issues. This includes developing skillful and experienced media spokespersons to convey scientific information in laymen friendly terms; tailoring vaccine communication plans pertinent to vaccine-specific or vaccine adverse event-specific issues; establishing multistakeholder networks at local, regional, and global levels to coordinate and refine messaging; active monitoring of vaccine knowledge, attitudes, practices (KAP), and related concerns and misinformation; and developing, implementing, and evaluating public communication interventions [23].

## 6. Regulatory Capacity and Workforce Constraints

While regulation is a critical enabler for sustainable immunization, many countries still lack mature and sufficiently resourced regulatory systems based on the WHO GBT [28]. This poses a risk for timely vaccine approval and safety surveillance, which may affect vaccination campaigns and undermine the sustainability of immunization programmes.

Limited investment in the regulatory workforce compounds this, resulting in long approval times due to under-resourcing in terms of sufficient numbers of adequately trained regulatory staff. Regulators also face the challenge of keeping up with technological advancements in the vaccine development landscape such as novel cell and gene-based vaccine platforms which may not fit in traditional regulatory frameworks. With these increasing demands, strengthening private and public partnerships and building an overall strong regulatory ecosystem with strong commitment to good regulatory science and internationally aligned regulatory practices are important features of smart regulation. While regulatory harmonization remains the ideal state, strong political commitment at regional and global levels is required to achieve this. Hence, regulatory convergence to minimize different requirements among NRAs through adoption of international regulatory standards, capacity building in expertise, application of reliance approaches and experience sharing by mature regulators is the more realistic and practical way forward. As NRAs progress in regulatory strengthening and convergence, regulatory reliance [29] and digitalization should continue to be implemented to minimize inefficiencies due to fragmented global and regional regulatory frameworks that negatively affect vaccine access and the sustainability of immunization programmes.

## 7. Regulation as a Pillar of Health System Resilience

**Figure 1:**
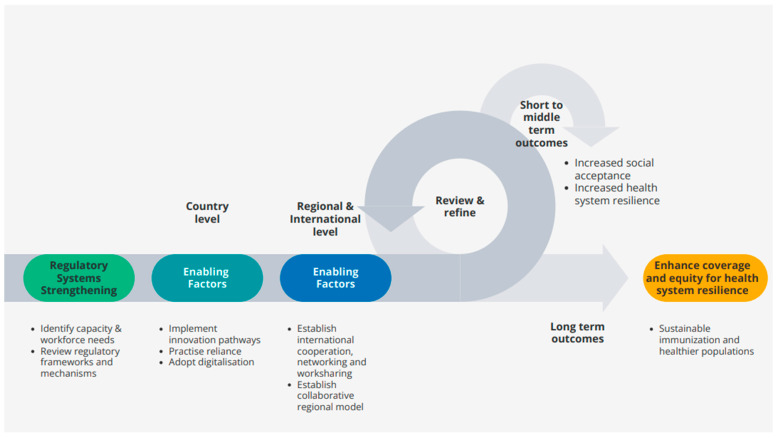
Regulatory systems strengthening and enabling factors to build health systems resilience.

Developing robust regulatory systems, building workforce capacity and strengthening local, regional, and global networks can enhance the resilience of national health systems by assuring timely access to safe, efficacious, and quality vaccines and fostering innovation tailored to local disease burdens during inter-pandemic periods (Figure 1). Strengthened regulatory capacity can also support surge capacity during public health emergencies where the need for the rapid scale up of production, distribution, and uptake of vaccines is critical. Assessing regulatory maturity and capability solely on approval timelines and other regulatory metrics overlooks the essential role that regulation plays in supporting effective, resilient and trustworthy health system functions. Regulation is not an isolated technical activity but an integral component of health systems, continuously shaping performance by safeguarding quality, ensuring accountability, enabling equitable access, and maintaining public confidence in health interventions. A narrow understanding of the role and scope of regulation risks obscuring the systemic value of strong, well-functioning regulatory institutions in health system resilience. Without this shift in perspective, regulatory strengthening risks being underfunded, reactive, and fragmented, perpetuating inequities in vaccine access and undermining sustainability in immunization programmes.

Strengthening regulatory capacity for sustainable immunization should instead be understood as a strategic investment in enhancing health systems. Re-framing regulation as a pillar of health system resilience is important to highlight its broader function in safeguarding public health and enabling timely access to vaccines. Integration of science, policy, regulation, and practice can be further advanced by working with policy makers and thought leaders across scientific, regulatory, economic, and public health fields to expedite vaccine innovation for global health needs.

## 8. Conclusions

Discussions on sustainable immunization frequently focus on financing and delivery mechanisms, yet it is equally vital to recognize vaccine regulation as a foundational pillar of health system resilience that underpins the long-term sustainability of immunization programmes. While strengthening regulation alone cannot address all the challenges associated with sustainable immunization, its role remains crucial, particularly in driving innovation, and supporting sustainable and efficient timely vaccine access and safety surveillance to strengthen social acceptance as well as supporting regional collaboration models. Regulatory systems intersect with multiple stages of vaccine development, manufacturing, distribution, and safety monitoring to ensure a high quality, safe, and efficient outcome across the immunization ecosystem. When integrated with investments in the health system infrastructure and actionable pathways (Table 1), efficient regulation can act as a multiplier, amplifying the impact of other strategies aimed at achieving long-term immunization sustainability.

## Figures and Tables

**Table 1:** Actionable pathways to integrate regulatory strengthening and health systems resilience.

Embed regulatory strengthening in national immunization strategies Invest in regulatory workforce development and digital tools Align regulatory frameworks and mechanisms with international standards Institutionalize reliance, work-sharing and international cooperation

## Data Availability

No new data were created or analyzed in this study.

## Abbreviations

The following abbreviations are used in this manuscript:

AVSSR	ASEAN Vaccine Security and Self-Reliance (AVSSR)
CEPI	Coalition for Epidemic Preparedness Innovation (CEPI)
DPT	Diphtheria, tetanus, and pertussis (DPT)
EMA	European Medicine Agency
EUA	Emergency Use Authorisation
FRP	Facilitated regulatory pathways
HSA	Health Sciences Authority
IA2030	Immunization Agenda 2030
MFDS	Ministry of Food and Drug Safety
PMDA	Pharmaceuticals and Medical Devices Agency
PQ	Pre-qualification
SDG	Sustainable Development Goals (SDGs)
US FDA	United States Food and Drug Administration
WHO	World Health Organization
